# Association between screen time and non-suicidal self-injury among adolescents: a compositional isotemporal substitution analysis

**DOI:** 10.3389/fpubh.2026.1737730

**Published:** 2026-03-11

**Authors:** Wenzhuo Xu, Hao Guo, Kele Jiang, Haiyan Shi, Sainan Wang, Xinmiao Tang, Zheng Hu, Mengting Man, Wenhua Ruan, Anyi Geng, Guangbo Qu, Zhihua Zhang

**Affiliations:** 1 Department of Epidemiology and Biostatistics, School of Public Health, Anhui Medical University, Hefei, Anhui, China; 2 Hefei 168 Highschool, Hefei, Anhui, China; 3 Department of Public Health and Health Administration, Clinical College of Anhui Medical University, Hefei, Anhui, China

**Keywords:** 24-h physical activity, adolescents, compositional isotemporal substitution model, non-suicidal self-injury, screen time

## Abstract

**Background:**

In recent years, the global incidence of Non-Suicidal Self-Injury (NSSI) has risen, posing a significant challenge in public health. Adolescents are the main group affected.

**Methods:**

A cross-sectional study was conducted using a self-administered questionnaire to collect data from 6,311 adolescents in Hefei, China. This study employed the Compositional Isotemporal Substitution Model (CISM, a statistical method that estimates health effects of replacing time in one behavior with another while accounting for the interdependent, compositional nature of 24-h time-use data) to examine the impact of Screen Time (ST), Non-Screen-based Sedentary Time (NSST), Physical Activity, and Sleep Time on NSSI among adolescents.

**Results:**

Compositional logistic regression analysis revealed that, relative to the remaining behavioral components, higher Light Physical Activity (LPA) (*p* = 0.016) and Sleep Time (*p* < 0.001) were associated with a reduced risk of NSSI, while higher ST (*p* < 0.001) and NSST (*p* < 0.001) time were associated with an increased risk of NSSI. CISM showed that replacing LPA with ST was linked to an elevated risk of NSSI, whereas substituting ST with LPA was associated with a reduced risk of the behavior.

**Conclusion:**

The findings highlight those reasonably allocating adolescents’ daily activities, reducing ST, can help lower the risk of NSSI among adolescents.

## 1 Introduction

Non-Suicidal Self-Injury (NSSI) is defined as the deliberate act of damaging one’s own body tissues without suicidal intent or social/cultural sanction (1). Epidemiological studies indicate that NSSI occurs across all age groups, with the highest prevalence among adolescents (2). NSSI increases the risk of adolescents developing anxiety, depression, emotional disorders, substance abuse, and other adverse psychological and behavioral outcomes (3, 4). The frequent occurrence and aggravation of NSSI behaviors in adolescents may cause individuals to develop habits toward injury, pain, fear and death, thereby increasing the possibility of having suicidal thoughts and attempting suicide (5). Beyond endangering individual safety, NSSI also undermines family harmony (6), school safety (7), social stability (8), and adolescent health development (9), imposing a heavy societal burden.

In recent years, the increase in NSSI among teenagers has coincided with a sharp increase in the use of online technologies, reliance on social media, and ownership of smartphones (10). Specifically, excessive Internet use may displace real-life social interactions, leading to emotional recognition disorders, reduced empathy and curiosity, impaired emotional regulation, and increased behavioral problems (11). The increase in Internet use is accompanied by an increase in Screen Time (ST). Increased ST is closely related to a variety of physical and mental health issues. For example, chronic diseases such as obesity, cardiovascular disease, as well as common psychological problems among adolescents, including depression, anxiety (12). Furthermore, excessive ST is associated with elevated Sedentary Behavior (SB), reduced physical activity, altered Sleep (SLP) patterns, and depressive symptoms, which may further heighten the risk of NSSI, suicidal thoughts, and behaviors (13).

In addition to ST, Physical activity (PA), SB, and SLP are also critical variables in exploring NSSI’s influencing factors. PA refers to any bodily movement increasing energy expenditure via skeletal muscle contraction, classified by intensity into Moderate-to-Vigorous intensity Physical Activity (MVPA), and Light-intensity Physical Activity (LPA) (14). MVPA includes both Moderate intensity Physical Activity (MPA) and Vigorous intensity Physical Activity (VPA). SB describes sitting, reclining, or lying awake with an energy expenditure of ≤ 1.5 METs, and can be further divided into Screen-based Sedentary Time (SST) and Non-Screen-based Sedentary Time (NSST) (15). SLP refers to a state of rest in which conscious activity decreases or ceases (16).

Within a fixed time frame, there is a dynamic balance between the increase and decrease of activity time, which has a chain effect on health. The traditional Isochronous Substitution Model (ISM) can quantify activity substitution effects but is hampered by critical limitations (spurious correlation and multicollinearity) that undermine its analytical validity. 24-h activity behavior data are compositional data, characterized by their definite and restrictive properties, and require specialized methods for analysis. Compositional Data Analysis (CoDA) eliminates the fixed-sum constraint through isometric log-ratio (ILR) transformation. Combined with traditional regression models, CoDA can quantify the health impact of activity time substitution, identify behavior patterns related to adverse health outcomes, and determine the optimal time balance point. Research based on Compositional Isochronous Substitution Model (CISM) has become a current hotspot.

In conclusion, the complexity and multifactorial nature of NSSI necessitates a more comprehensive perspective that integrates multiple behaviors throughout the day to deeply explore its relationship with adolescent mental health. By employing advanced statistical methods, we can more scientifically quantify the impact of different activity behaviors on NSSI, thereby providing theoretical support and practical guidance for the development of targeted intervention strategies.

## 2 Materials and methods

### 2.1 Study participants

In this study, data were collected from April to June 2024 in four districtsfour districts (Yaohai District, Luyang District, Shushan District, and Baohe District) and Chaohu City (a county-level city) under the jurisdiction of Hefei City were selected. Two middle schools and two high schools were randomly selected from each district, totaling 20 schools. Using a stratified cluster sampling method, four classes from each of the first and second grades were randomly selected from each school, with a total of 7,081 students included in the study. Due to missing or incomplete basic demographic information (*n* = 135) or survey data (*n* = 635), the final number of valid questionnaires included in the analysis was 6,311, yielding an effective response rate of 89.13%. Supplementary Figure 1 shows the flowchart of the inclusion of participants.

### 2.2 Study content and methods

#### 2.2.1 General information

A self-designed socio-demographic questionnaire was administered to collect general personal information from the participants, including grade, gender, age, height, weight, only child status, boarding student or not, academic performance, experience of staying behind, parents’ education level, and per capita family income.

#### 2.2.2 Physical activity

The Chinese version of the International Physical Activity Questionnaire Short Form (IPAQ-SF) was used to assess the physical activity levels of the study subjects (17). This questionnaire is based on participants’ self-reported activity levels over the past 7 days and contains seven items. It is designed to collect the daily duration (in minutes) and weekly frequency (in days) of walking, VPA, MPA, LPA, and SB time. Qu and Lis (18) study on the reliability and validity of the IPAQ-SF showed that the intraclass correlation coefficients of various physical activity items all exceeded 0.7.

#### 2.2.3 Sleep

SLP time was assessed using the translated and validated Chinese version of the Pittsburgh Sleep Quality Index (PSQI), a widely recognized measure of SLP quality (19). This scale evaluates individuals’ SLP quality over the past month, with a specific focus on SLP duration. Liu (20) validated the reliability of this scale in China, with a Cronbach’s *α* coefficient of 0.84.

#### 2.2.4 Screen time

The design of the ST survey questionnaire in this study was adapted from the measurement content of ST in the Youth Risk Behavior Survey (YRBS). This questionnaire is a widely used tool both domestically and internationally, having undergone multiple validations. The reliability and validity of this part of the questionnaire were tested by Tang et al. using Cronbach’s α coefficient, with an internal consistency α coefficient of 0.847, indicating high reliability (21). It assessed students’ ST over the past month, including the following aspects:

Types of screens: TV, computer, mobile phone, tablet, electronic game consoles, e-book readers (e.g., Kindle), or other electronic devices.
ST: ST on school days was multiplied by 5, and ST on weekends was multiplied by 2. The total was then summed and divided by 7 to calculate the average daily ST over the past week.

#### 2.2.5 Non-suicidal self-injury

The study adopted the Chinese revised version of the Functional Assessment of Self-Mutilation (C-FASM), originally the Functional Assessment of Self-Mutilation (FASM), to evaluate the methods, frequency, and functions of NSSI among adolescents. This scale has been tested among Chinese adolescents and demonstrates good reliability and validity, with a Cronbach’s *α* coefficient of 0.81 (22). The 10-item assessment component measures NSSI behaviors and their frequency over the past year. A five-point Likert scale was used to score the frequency of each NSSI behavior: 0 times (0), 1 time (1), 2–5 times (2), 6–10 times (3), and ≥ 11 times (4). Higher scores corresponded to higher frequencies of NSSI behaviors (23).

### 2.3 Statistical analysis

#### 2.3.1 Compositional data analysis

To conduct compositional regression analysis, Isometric Log Ratio (ILR) transformations were applied to map the compositional data into real space. This process reduces data dimensionality and enables the use of compositional data in standard statistical models. Since zero values cannot be transformed into log-ratio, the zero values in the co were imputed using the log-ratio expectation maximization algorithm (24). The structure of the ILR transformation is determined by Sequential Binary Partition (SBP), which constructs the first pivot coordinate to capture the relative information between a dominant activity (numerator) and the remaining activities (denominator) (25). In this study, five ILR transformation results were obtained by placing each of the five activity behaviors (MVPA, LPA, SLP, ST, NSST) in the numerator. For example, MVPA is transformed as follows:


$$\mathrm{IL}R_{1\_\text{MVPA}}=\sqrt{4/5}\ln\frac{\text{MVPA}}{\sqrt[{4}]{\mathrm{LPA}*\mathrm{SLP}*\mathrm{ST}*\text{NSST}}}$$

$$\mathrm{IL}R_{2\_\text{MVPA}}=\sqrt{3/4}\ln\frac{\mathrm{LPA}}{\sqrt[{3}]{\mathrm{SLP}*\mathrm{ST}*\text{NSST}}}$$

$$\mathrm{IL}R_{3\_\text{MVPA}}=\sqrt{2/3}\ln\frac{\mathrm{SLP}}{\sqrt[{2}]{\mathrm{ST}*\text{NSST}}}$$

$$\mathrm{IL}R_{4\_\text{MVPA}}=\sqrt{1/2}\ln\frac{\mathrm{ST}}{\sqrt[{1}]{\text{NSST}}}$$

Since only the first ILR coordinate captures all relevant information about a specific behavior, each of the five behaviors was placed separately to examine the effect of temporal changes in that behavior relative to the others on the dependent variable. A compositional logistic regression model was constructed, with the five activity behavior coordinates transformed by ILR serving as independent variables and NSSI as the dependent variable. Covariates such as gender, grade, BMI, academic performance, and other general information were controlled for in the model. The predicted Odds of MVPA as the first order behavior model were:


$$\begin{array}{l} \hat{\text{Odds}}(\text{MVPA},\mathrm{LPA},\mathrm{SLP},\mathrm{ST},\text{NSST})=\\ \exp(\begin{array}{l} \hat{\beta_{0}}+\hat{\gamma_{1}}*\sqrt{4/5}\ln\frac{\text{MVPA}}{\sqrt[{4}]{\mathrm{LPA}*\mathrm{SLP}*\mathrm{ST}*\text{NSST}}}+\\ \hat{\gamma_{2}}*\sqrt{3/4}\ln\frac{\mathrm{LPA}}{\sqrt[{3}]{\mathrm{SLP}*\mathrm{ST}*\text{NSST}}}+\\ \hat{\gamma_{3}}*\sqrt{2/3}\ln\frac{\mathrm{SLP}}{\sqrt[{2}]{\mathrm{ST}*\text{NSST}}}+\\ \hat{\gamma_{4}}*\sqrt{1/2}\ln\frac{\mathrm{ST}}{\sqrt[{1}]{\text{NSST}}}+\hat{\beta }*\text{covariate} \end{array}) \end{array}$$

#### 2.3.2 Compositional isotemporal substitution model

Based on the fitted compositional logistic regression model, the effect of “one-to-one” substitution of MVPA, LPA, SLP, ST, and NSST with equal amounts of time on the risk of NSSI was analyzed, while maintaining a constant total time (e.g., replacing 10 min of ST with 10 min of LPA). Consistent with relevant research (26), activities were substituted in units of 10 min. The difference between the time distribution after reallocation and the predicted value of the original time distribution was calculated based on the fitted model. The change in NSSI risk before and after time reallocation was expressed as an Odds Ratio (*OR*) and calculated as follows, the *OR* is the predicted value calculated based on the compositional logistic regression model.


$$\text{OR}=\frac{\hat{\text{Odds}}(\bar{\text{MVPA}}-10,\bar{\mathrm{LPA}},\bar{\mathrm{SLP}},\bar{\mathrm{ST}}+10,\bar{\text{NSST}})}{\hat{\text{Odds}}(\bar{\text{MVPA}},\bar{\mathrm{LPA}},\bar{\mathrm{SLP}},\bar{\mathrm{ST}},\bar{\text{NSST}})}$$

## 3 Results

### 3.1 The demographic characteristics of the research subjects

A total of 6,311 participants were enrolled in this study, with a mean age of 14.64 ± 1.68 years. Among them, 3,443 were males (54.56%) and 2,868 were females (45.44%); 3,234 were junior high school students (51.24%) and 3,077 were senior high school students (48.76%). The majority of participants had a normal body mass index (BMI), accounting for 54.40%, followed by 1829 underweight participants (28.98%). A total of 2069 participants were only children, accounting for 32.78, and 81.16% of the participants were non-boarding students. In terms of academic performance, 1894 participants achieved average grades (30.01%) and 2029 attained upper-average grades (32.15%). For paternal educational level, the largest proportion of participants had fathers with junior high school education or below (35.21%); similarly, the majority of participants had mothers with junior high school education or below (44.11%). In addition, 3,938 participants (62.40%) had fathers who had never worked away from home for a long time, and 5,129 participants (81.27%) had mothers who had never worked away from home for a long time.

### 3.2 Screen time

The Mann–Whitney U test and Kruskal-Wallis H test were employed to examine the distribution characteristics of ST among adolescents across different genders and grades. Mann–Whitney U test was used to compare the differences between genders, and no significant differences were observed between boys and girls across all grades (*p* > 0.05). The Kruskal-Wallis H test revealed significant differences in ST among different grades (*p* < 0.001). Pairwise comparisons revealed that there were no significant differences in ST between junior high school Grade 1 and Grade 2 students (*p* > 0.05) or between senior high school Grade 1 and Grade 2 students (*p* > 0.05), as shown in Table 1.

**Table 1:** Distribution characteristics of ST.

Grade	Boy		Girl		Total	
	*N*1	Median (*P*_25_-*P*_75_)	*N*2	Median (*P*_25_-*P*_75_)	*N*	Median (*P*_25_-*P*_75_)
A^a^	928	239.64 (102.86–335.71)^cd^	693	242.86 (105.71–321.43)^cd^	1,621	240.00 (103.57–327.50)^cd^
B^b^	871	240.00 (102.86–344.29)^cd^	742	262.86 (105.36–377.14)^d^	1,613	252.86 (102.86–359.64)^cd^
C^c^	830	273.93 (137.14–388.57)^ab^	698	276.46 (150.71–400.00)^a^	1,528	274.29 (141.43–394.29)^ab^
D^d^	814	275.39 (154.29–423.57)^ab^	735	290.00 (162.86–414.29)^ab^	1,549	282.86 (158.57–418.57)^ab^
Total	3,443	258.57 (124.29–370.00)	2,868	267.14 (128.57–377.14)	6,311	264.29 (128.57–374.39)

A: Junior high school Grade 1; B: Junior high school Grade 2; C: Senior high school Grade 1; D: Senior high school Grade 2. ^b–d^represents that there were significant differences between group a and groups ^b–d^in pairwise comparisons, *p* < 0.05.

### 3.3 Non-suicidal self-injury

Among the 6,311 students surveyed, the prevalence of NSSI was 22.74% in boys and 32.53% in girls, with girls exhibiting a significantly higher rate than boys (*p* < 0.001). Significant differences in NSSI prevalence were observed across various demographic characteristics, including grade, only child status, living situation, academic performance, mother’s education level, and parents working outside the home status (all *p* < 0.05), as shown in Table 2.

**Table 2:** Comparison of NSSI with different demographic characteristics.

Variable	No NSSI (%)	NSSI (%)	*x* ^2^	*p* value
Gender			75.74	<0.001
Boy	2,660 (77.26)	783 (22.74)		
Girl	1935 (67.47)	933 (32.53)		
Grade			19.32	<0.001
Junior high	2,277 (70.41)	957 (29.59)		
Senior high	2,318 (75.33)	759 (24.67)		
BMI			2.19	0.533
Underweight	1,348 (73.70)	481 (26.30)		
Normal	2,444 (72.52)	926 (27.48)		
Overweight	569 (73.14)	209 (26.86)		
Obese	234 (70.06)	100 (29.94)		
Only child			4.37	0.037
Yes	1,514 (74.48)	528 (25.52)		
No	3,050 (71.98)	1,187 (28.02)		
Living situation			7.281	0.007
Boarding student	903 (75.95)	286 (24.05)		
Day student	3,692 (72.08)	1,430 (27.92)		
Academic performance			31.163	<0.001
Lower class	287 (66.13)	147 (33.87)		
Lower middle class	865 (68.92)	390 (31.08)		
Middle class	1,389 (73.34)	505 (26.66)		
Upper middle class	1,510 (74.42)	519 (25.58)		
Upper class	544 (77.83)	155 (22.17)		
Father’s educational level			5.746	0.332
Junior high school or below	1,622 (73.00)	600 (27.00)		
Senior high school	1,124 (73.51)	405 (26.49)		
Secondary vocational school / Technical school	306 (68.61)	140 (31.39)		
Junior college	623 (72.72)	239 (27.73)		
Bachelor’s degree	757 (72.93)	281 (27.07)		
Graduate degree or above	163 (76.17)	51 (23.83)		
Mather’s educational level			12.24	0.032
Junior high school or below	2041 (73.31)	743 (26.69)		
Senior high school	921 (72.92)	342 (27.08)		
Secondary vocational school / Technical school	324 (66.94)	160 (33.06)		
Junior college	594 (72.09)	230 (27.91)		
Bachelor’s degree	610 (74.12)	213 (25.88)		
Graduate degree or above	105 (78.55)	28 (21.05)		
Per capita family income (CNY)			8.19	0.224
<1 ten thousand	353 (73.54)	127 (26.46)		
1 ~ 2.9 ten thousand	847 (72.46)	322 (27.54)		
3 ~ 4.9 ten thousand	1,064 (71.7)	420 (28.3)		
5 ~ 6.9 ten thousand	859 (75.95)	272 (24.05)		
7 ~ 8.9 ten thousand	477 (72.82)	178 (27.18)		
9 ~ 10.9 ten thousand	334 (70.76)	138 (29.24)		
≥11 ten thousand	661 (71.85)	259 (28.15)		
Father’s long-term work away from home			26.28	<0.001
Never	2,955 (75.04)	983 (24.96)		
Yes	1,640 (69.11)	733 (30.89)		
Mather’s long-term work away from home			11.92	<0.001
Never	3,782 (73.74)	1,347 (26.26)		
Yes	813 (68.78)	369 (31.22)		

NSSI, Non-Suicidal Self-Injury; CNY, Chinese Yuan.

Supplementary Table 1 presents the results of the comparison of NSSI detection rates among adolescents in different ST groups. Overall, adolescents who spent less than 2 h per day on screen activities exhibited a significantly lower prevalence of NSSI compared to those who spent more than 2 h per day (22.1% vs. 28.81%, *p* < 0.001). Specifically, the prevalence of NSSI was significantly lower among boys with <2 h of daily screen time than among those with ≥2 h (18.5% vs. 24.1%, *p* = 0.001), and a similar pattern was observed among girls (26.5% vs. 34.4%, *p* < 0.001).

### 3.4 Time distribution of 24-hour activity behaviors

#### 3.4.1 The component geometric means of 24-h activity

The geometric means of the 24-h activity time used by respondents and its proportion within the 24-h period. For both boys and girls, the order of activity components by their percentage contribution to the 24 h, from highest to lowest, was: SLP > NSST > ST > MVPA > LPA. The detailed data are presented in the Supplementary Table 2.

#### 3.4.2 NSSI differences in temporal distribution

**Figure 1:**
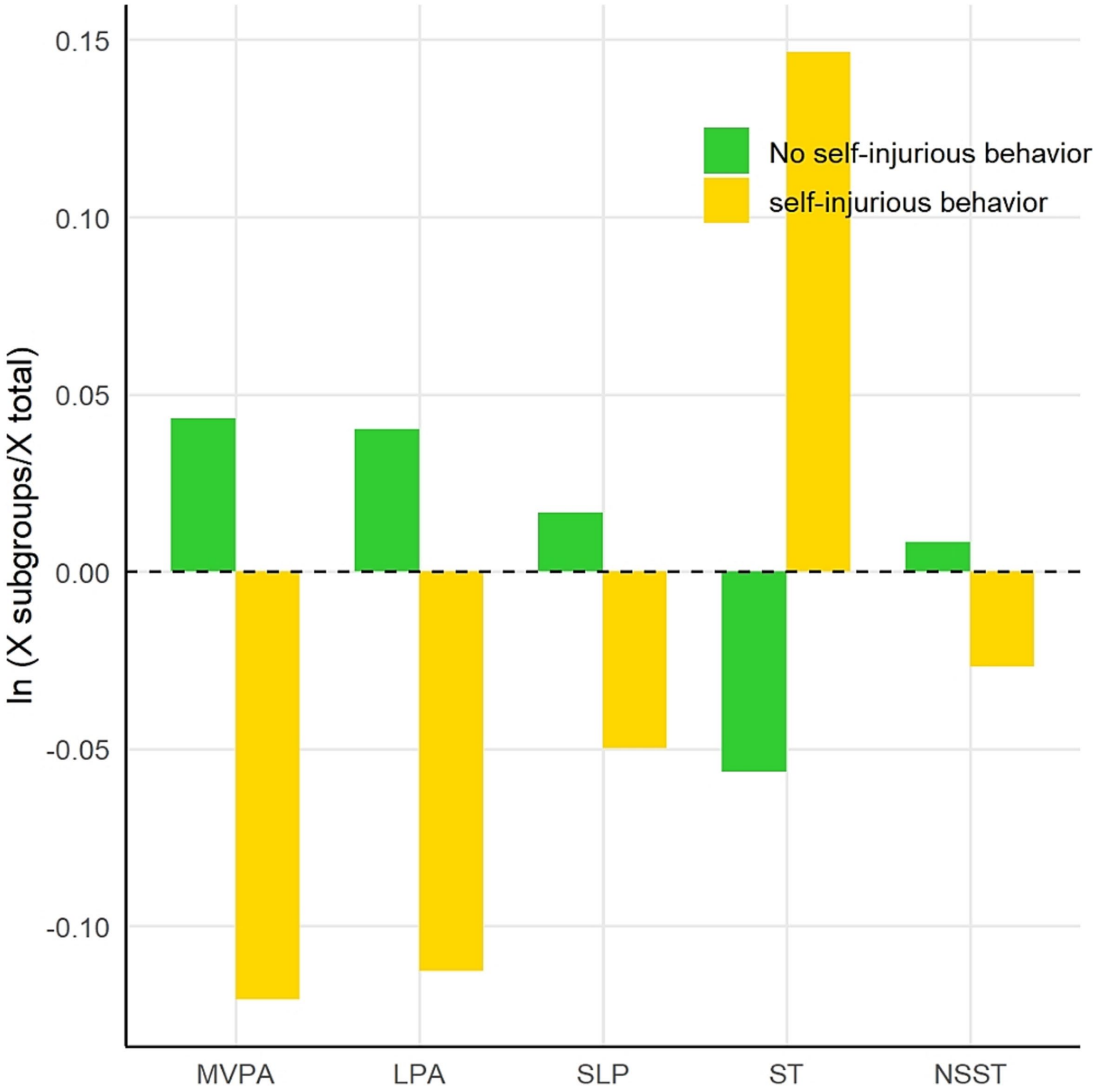
Bar chart of geometric means of five activity behaviors in NSSI subgroups.

A geometric mean bar graph was constructed based on the component geometric mean of the five activity behaviors over 24 h, with presence or absence of NSSI as the subgroup. The log ratio ln (x subgroup / x population) was used as the ordinate, and the five activity behaviors (MVPA, LPA, SLP, ST, and NSST) were plotted along the abscissa. The results showed that among the five activity behaviors, adolescents with NSSI spent a higher proportion of time in MVPA, LPA, SLP, and NSST, and a lower proportion in ST, while adolescents without NSSI spent a higher proportion in ST. The proportion of time spent in MVPA, LPA, and SLP was relatively low overall, as shown in Figure 1.

#### 3.4.3 Comparison of NSSI detection rates with different intensity physical activity and SLP duration

In accordance with the Physical Activity Guidelines for Chinese Children and Adolescents, the International 24-Hour Movement Guidelines for Children and Youth, and the Canadian Youth Physical Activity Guidelines, participants were categorized based on their behavior types, and their NSSI status was analyzed. The results indicated that adolescents with varying durations of MVPA, LPA, and SLP exhibited different NSSI statuses. The detailed data are presented in Supplementary Table 3.

### 3.5 Impact of replacing different types of activities on NSSI

#### 3.5.1 Compositional logistic regression model

The results of the logistic regression analysis examining the relationship between each 24-h activity behavior and NSSI in adolescents are presented in Table 3. The findings indicate that an increase in LPA time is associated with a reduced risk of NSSI among adolescents (OR = 0.942, 95% CI = 0.897–0.989, *p* = 0.016). Similarly, an increase in SLP time is associated with a reduced risk of NSSI (OR = 0.674, 95% CI = 0.590–0.767, *p* < 0.001). Conversely, an increase in ST is associated with an elevated risk of NSSI (OR = 1.344, 95% CI = 1.258–1.440, *p* < 0.001). Additionally, an increase in NSST is associated with a higher risk of NSSI (OR = 1.204, 95% CI = 1.111–1.306, *p* < 0.001).

**Table 3:** Compositional logistic regression results of 24-h activity behaviors and NSSI.

Type of activity behavior	Regression coefficient	SE	*p* value	OR (95% CI)
ILR MVPA/LPA*SLP*ST*NSST	−0.026	0.021	0.224	0.974 (0.934, 1.016)
ILR LPA/MVPA*SLP*ST*NSST	−0.060	0.025	0.016	0.942 (0.897, 0.989)
ILR SLP/MVPA*LPA*ST*NSST	−0.395	0.067	<0.001	0.674 (0.590, 0.767)
ILR ST/MVPA*LPA*SLP*NSST	0.296	0.035	<0.001	1.344 (1.258, 1.440)
ILR NSST/MVPA*LPA*SLP*ST	0.186	0.041	<0.001	1.204 (1.111, 1.306)

SE, Standard error; OR, Odds Ratio; CI, Confidence interval; MVPA, Moderate-to-vigorous physical activity; LPA, Low-intensity physical activity; ST, Screen time; NSST, Non-sedentary screen time; SLP, Sleep.

#### 3.5.2 Compositional isotemporal substitution model

Using the CISM, the *ORs* and 95% *CI*s for 10, 20, and 30 min of mutual substitution between MVPA, LPA, SLP, ST, and NSST on adolescent NSSI were analyzed. The findings indicated that substituting LPA with other behaviors was associated with an increased risk of NSSI in adolescents, regardless of the substitution time (10, 20, or 30 min), with the corresponding *OR* values gradually increasing as the substitution time extended. Conversely, substituting ST with other behaviors was associated with a reduced risk of NSSI in adolescents, with the corresponding *OR* values decreasing as the substitution time increased. The substitutions of LPA and ST for 10, 20, and 30 min had statistically significant effects on NSSI in adolescents. Specifically, substituting ST for LPA was associated with an increased risk of NSSI (10 min: OR = 1.029, 95% CI = 1.021–1.036; 20 min: OR = 1.071, 95% CI = 1.050–1.092; 30 min: OR = 1.184, 95% CI = 1.120–1.251). Conversely, substituting LPA for ST was associated with a decreased risk of NSSI (10 min: OR = 0.977, 95% CI = 0.972–0.983; 20 min: OR = 0.958, 95% CI = 0.948–0.968; 30 min: OR = 0.940, 95% CI = 0.928–0.953). Table 4 shows in detail the results of the analysis of the effect of substituting different activity behaviors for each other on NSSI.

**Table 4:** Expected *OR* values and 95% *CI*s for NSSI after 10 min, 20 min, 30 min substitution of 24-h activity behaviors.

	MVPA↓	LPA↓	SLP↓	ST↓	NSST↓
MVPA↑		**1.015 (1.006, 1.025)**	1.001 (0.997, 1.005)	**0.987 (0.983, 0.991)**	**0.991 (0.987, 0.995)**
		**1.044 (1.021, 1.068)**	1.002 (0.995, 1.009)	**0.975 (0.968, 0.981)**	**0.983 (0.976, 0.989)**
		**1.142 (1.078, 1.210)**	1.004 (0.994, 1.014)	**0.963 (0.954, 0.972)**	**0.975 (0.965, 0.984)**
LPA↑	**0.991 (0984, 0.999)**		**0.991 (0.985, 0.997)**	**0.977 (0.972, 0.983)**	**0.981 (0.976, 0.987)**
	0.988 (0.972, 1.004)		**0.985 (0.975, 0.995)**	**0.958 (0.948, 0.968)**	**0.966 (0.956, 0.975)**
	0.991 (0.965, 1.018)		**0.981 (0.967, 0.995)**	**0.940 (0.928, 0.953)**	**0.952 (0.939, 0.965)**
SLP↑	1.001 (0.996, 1.005)	**1.015 (1.007, 1.023)**		**0.986 (0.985, 0.988)**	**0.990 (0.989, 0.992)**
	1.003 (0.992, 1.015)	**1.043 (1.022, 1.064)**		**0.959 (0.955, 0.963)**	**0.971 (0.967, 0.976)**
	1.003 (0.992, 1.015)	**1.043 (1.022, 1.064)**		**0.959 (0.955, 0.963)**	**0.971 (0.967, 0.976)**
ST↑	**1.014 (1.010, 1.019)**	**1.029 (1.021, 1.036)**	**1.014 (1.012, 1.015)**		**1.004 (1.003, 1.005)**
	**1.030 (1.020, 1.042)**	**1.071 (1.050, 1.092)**	**1.028 (1.025, 1.030)**		**1.007 (1.005, 1.009)**
	**1.051 (1.030, 1.073)**	**1.184 (1.120, 1.251)**	**1.041 (1.037, 1.045)**		**1.010 (1.007, 1.013)**
NSST↑	**1.010 (1.006, 1.015)**	**1.025 (1.017, 1.032)**	**1.010 (1.008, 1.011)**	**0.996 (0.995, 0.997)**	
	**1.023 (1.012, 1.034)**	**1.063 (1.042, 1.084)**	**1.020 (1.017, 1.023)**	**0.991 (0.990, 0.993)**	
	**1.040 (1.019, 1.061)**	**1.171 (1.108, 1.237)**	**1.029 (1.025, 1.034)**	**0.987 (0.984, 0.989)**	

MVPA, Moderate-to-vigorous physical activity; LPA, Low-intensity physical activity; ST, Screen time; NSST, Non-sedentary screen time; SLP, Sleep. The three lines corresponding to each type of physical activity, from top to bottom, are 10, 20, and 30 min. Bolded values: the statistical results are significant, *p* < 0.05.

**Figure 2:**
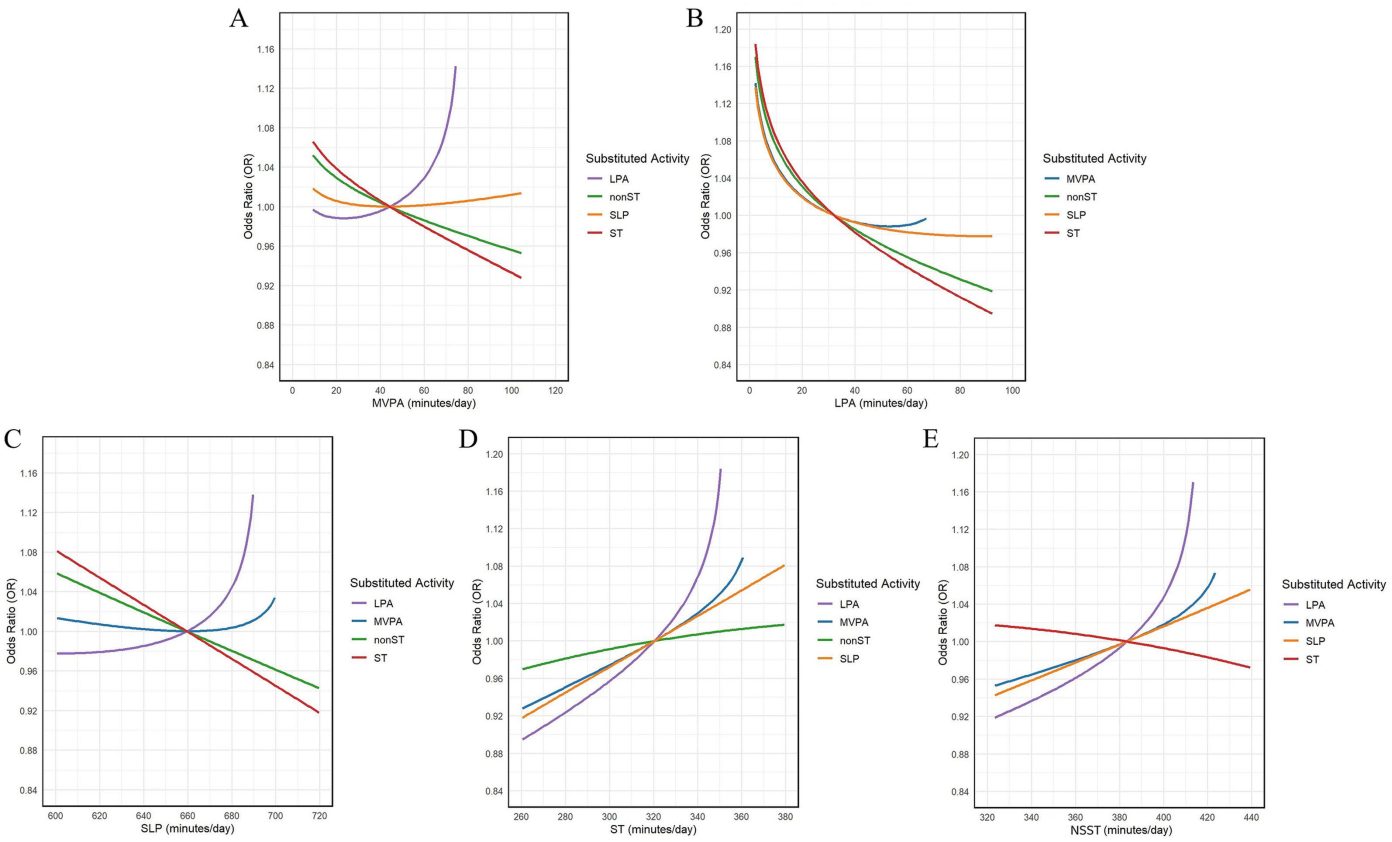
Trend of NSSI expected *OR* changes during time reallocation. **(A)** Changes in expected *OR* of NSSI due to MVPA substitution for other behaviors; **(B)** Changes in expected *OR* of NSSI due to LPA substitution for other behaviors; **(C)** Changes in expected *OR* of NSSI due to SLP substitution for other behaviors; **(D)** Changes in expected *OR* of NSSI due to ST substitution for other behaviors; **(E)** Changes in expected *OR* of NSSI due to NSST substitution for other behaviors.

Due to the non-negativity constraint of compositional data, the replacement times for MVPA and LPA did not exceed their respective geometric means. Figures 2 illustrates the changes in the expected OR values of NSSI during the time redistribution process. In these figures, the intersection points represent the geometric means of the corresponding behavior times. The left side of the intersection points shows the impact of substituting the behavior with the other four behaviors on the expected OR values of NSSI, while the right side shows the impact of substituting the other four behaviors with the behavior in question.

## 4 Discussion

### 4.1 NSSI in adolescents

This study found that the prevalence of NSSI among adolescents in the past year was 27.19%, with the three most common behaviors being intentional self-biting (e.g., mouth or lips), intentional hair-pulling, and punching or hitting hard objects with fists or heads. Adolescent NSSI prevalence varies by region. For instance, Tatnell et al. (27) reported a prevalence of 9.4% among 12 to 15-year-old adolescents in Australia, which is lower than our findings. In contrast, a cross-sectional study involving 12,068 adolescents across 11 European countries found a prevalence of 27.6% (28), while Plener et al. (29) reported a prevalence of 25.6% among 665 ninth-grade students in Germany. These results are generally comparable to our study. However, direct comparisons may be challenging due to differences in regional cultures among study populations. Another study reported a prevalence of 38.9% for NSSI among Chinese adolescents, with biting (62.3%, *n* = 230) being the most common method, followed by scratching the skin (58.8%, *n* = 217) (30). Two potential reasons may account for the discrepancies. First, variations in measurement instruments, such as the use of different scales, may contribute to differences in NSSI prevalence. Second, the cutoff value for defining NSSI may be a significant factor. For example, some studies defined individuals who engaged in three or more NSSI behaviors within the past year as having NSSI (31), whereas our study classified individuals who engaged in one or more NSSI behaviors as having NSSI behaviors.

Our study found that the incidence of NSSI was significantly higher in girls than in boys, consistent with previous research (32). This difference may be attributed to differences in physical growth, psychological development stages, and hormone levels between males and females, which collectively contribute to females entering puberty earlier and encountering emotional challenges and psychological disorders sooner, potentially making them more susceptible to NSSI (33). The prevalence of NSSI varied among adolescents in different grades, with junior high school students (29.59%) exhibiting a higher rate than senior high school students (24.67%). This aligns with Plener et al. (34) observation that NSSI peaks in early and middle adolescence. This may be due to the relative immaturity of adolescents in this stage, their propensity for impulsive behaviors, and their lack of clear understanding of the potential negative consequences of NSSI. Adolescents with lower academic performance had a higher detection rate of NSSI, possibly due to increased academic pressure and self-denial, leading to heightened emotional problems and NSSI. Targeted interventions are needed for these high-risk groups: schools and families should collaborate to provide mental health courses enhancing emotional regulation and reducing impulsivity, offer personalized academic guidance and counseling to alleviate pressure and self-denial, and strengthen public health education to raise awareness of NSSI harms and promote healthy coping styles for negative emotions.

### 4.2 ST in adolescents

According to guidelines from the American Academy of Pediatrics (AAP) and the Activity Guidelines for Children and Adolescents in China, adolescents are recommended to limit their ST to less than 2 h per day (35). In this study, the overall detection rate of ST ≥ 2 h per day among middle school students was 75.80%. Specifically, the detection rate among girls with ST ≥ 2 h per day was 76.20%, slightly higher than that among boys (75.50%). These rates were higher than those reported in previous studies (36), this discrepancy may be attributed to the inclusion of LCD teaching tool usage in classroom ST statistics in this survey, which could significantly increase the total ST. The prevalence of online education has led teenagers to use electronic devices not only during class but also after class for homework and independent learning, further extending their ST.

This study found that the detection rate of NSSI was significantly higher among adolescents with ST ≥ 120 min per day compared to those with ST < 120 min (28.81% vs. 22.1%). This finding was consistent across both boys and girls and aliens with previous research. While moderate ST can provide adolescents with beneficial health information, academic resources, and social connections (37), excessive ST may have detrimental effects on mental health. Adolescents may be exposed to false or misleading information, inappropriate media content, or harmful online content that promotes NSSI behaviors (38). Given the underdevelopment of the adolescent prefrontal cortex, they may lack the ability to critically evaluate such information. This cognitive strain can induce stress, potentially leading to NSSI as a coping mechanism. Therefore, it is crucial for parents and schools to guide adolescents in managing their ST and to provide education and training to enhance their ability to discern online information, thereby mitigating the impact of false or misleading content.

### 4.3 Characteristics of 24-h activity behavior time in adolescents

The study results indicated that, in terms of the proportion of 24-h activity behavior time, the order was as follows: SLP, NSST, ST, MVPA, and LPA. The results showed that SLP and sedentary time (including ST and NSST time) accounted for a large proportion of adolescents’ daily activities, while MVPA and LPA time were relatively insufficient. This finding is consistent with domestic and international studies, suggesting that adolescents generally have insufficient physical activity, particularly with MVPA time significantly lower than the international guideline recommendation of at least 60 min per day (39). This pattern of time allocation may be related to academic pressure, the prevalence of electronic devices, and changes in the social environment. In the school environment, long hours of classroom learning and homework after school also lead to a significant increase in sedentary time among adolescents.

The geometric mean bar chart results revealed differences in the distribution of the five activities between boys and girls. Boys spent more time in MVPA than girls, while girls spent more time in ST and NSST. This difference may be related to gender role perception, sociocultural factors, and adolescents’ personal interests and habits. Boys are generally more inclined to participate in physical activities, while girls may spend more time in SB, such as studying or using electronic devices. Moreover, compared to the overall group, adolescents with NSSI spent more time on ST and relatively less time on MVPA, LPA, and SLP. This finding is consistent with existing studies suggesting that SB, especially ST, may be associated with NSSI (40). Prolonged use of electronic devices may lead to social isolation, impaired SLP quality, and emotional problems, thereby increasing the risk of NSSI. Additionally, adolescents without NSSI behaviors spent a higher proportion of time in MVPA and LPA, suggesting that physical activity may have a protective effect on mental health. Practically, schools should extend and improve physical education classes, offer diverse physical activity programs for different genders and interests, and reduce academic burden to free up activity time. Families should encourage outdoor and light-intensity activities (e.g., walking, housework) and limit sedentary time, especially ST. Public health departments can launch campaigns to raise awareness of balanced 24-h activities, guiding adolescents to establish healthy habits.

### 4.4 Impact of 24-hour activity time substitution on NSSI in adolescents

The results of the compositional logistic regression model showed that increased LPA and SLP time were associated with a decreased risk of NSSI, while increased ST and NSST were associated with an increased risk of NSSI. Consistent with previous findings, these results suggest that physical activity and adequate SLP have protective effects on mental health, while SB, particularly ST, may increase the risk of NSSI (41). Although the *OR* for LPA was relatively low (*OR* = 0.942, 95% *CI* = 0.897–0.989), the positive effect of LPA on mental health may be related to the relaxation and emotion regulation effects of these activities. Khazaie et al. (40) have demonstrated that even low-intensity physical activities, such as walking or light housework, can relieve stress and improve mood, thereby reducing the risk of NSSI. Increased SLP duration was significantly associated with a reduced risk of NSSI, highlighting the essential role of adequate SLP in adolescent mental health. Insufficient SLP can lead to decreased emotion regulation ability and impaired cognitive function, which may increase the risk of NSSI (42). Increased ST and NSST were significantly associated with an increased risk of NSSI (ST: OR = 1.344, 95% CI = 1.258–1.440; NSST: OR = 1.204, 95% CI = 1.111–1.306). This finding aligns with several studies showing that SB, especially ST associated with the use of electronic devices (such as smartphones, computers, tablets, etc.), may increase the risk of NSSI by leading to social isolation, emotional problems, and reduced opportunities for face-to-face socialization (43). Moreover, increased ST is often accompanied by decreased SLP quality. Research has shown that the blue light emitted by electronic devices inhibits melatonin secretion and disrupts the SLP cycle in adolescents, resulting in insufficient or poor-quality SLP (44). Similarly, increased NSST was associated with an increased risk of NSSI, possibly due to the decreased physical activity time and its indirect negative impact on mental health.

CISM analysis further elucidated the effects of mutual substitution among the five different activity behaviors on the risk of NSSI. The results of 10 min, 20 min, and 30 min “one-on-one” behavioral substitutions consistently showed that substituting LPA for other behaviors was associated with an increased risk of NSSI in adolescents. Conversely, the risk of NSSI was significantly reduced when LPA replaced other active behaviors. Substituting other behaviors for ST was associated with a reduced risk of NSSI, while substituting ST for other active behaviors significantly increased the risk of NSSI. These findings suggest that reducing ST and increasing LPA time may be an effective strategy to mitigate the risk of NSSI. The substitution effects were asymmetrical and more pronounced with increasing substitution time, likely due to differences in the relative contributions of the five activity behaviors over 24 h. For instance, LPA accounts for a smaller fraction of the day compared to ST, so a 10-min reduction in ST has a relatively minor impact on the entire day, whereas a 10-min reduction in LPA has a more significant effect. When MVPA replaced LPA and SLP, the risk of NSSI tended to increase. However, when MVPA replaced ST and NSST, the risk of NSSI tended to decrease. This indicates that, despite the positive health effects of MVPA, it cannot fully compensate for the negative effects of excessive ST and insufficient SLP. Therefore, when devising intervention measures, it is crucial to consider the balance among different activity behaviors. When SLP time replaced ST and NSST time, the risk of NSSI was significantly reduced. Conversely, when SLP time replaced MVPA and LPA time, the risk of NSSI tended to increase. This result further underscores the importance of adequate SLP for mental health and suggests that excessive reductions in physical activity time should be avoided when increasing SLP time.

From an intervention perspective, our findings suggest practical strategies for schools and parents. Replacing ST with LPA or NSST may represent a feasible approach to reduce NSSI risk among adolescents. Unlike VPA, LPA requires minimal equipment and can be easily integrated into daily school schedules or family routines. Similarly, non-screen sedentary activities (e.g., reading, board games) offer accessible alternatives that maintain engagement while eliminating screen exposure. Schools could implement policies limiting screen use during breaks while providing structured LPA opportunities, while parents might establish screen-free zones or times at home. These substitutions may be more sustainable than complete screen abstinence, potentially offering a pragmatic public health approach to adolescent mental health promotion.

### 4.5 Limitations

In this study, the Compositional Isotemporal Substitution Model (CISM) was used to evaluate the impact of time allocation among different activity behaviors on the risk of NSSI. By applying Compositional Data Analysis (CoDA), the limitations of traditional isochronous substitution methods were addressed, and the problems of spurious correlation and multicollinearity were eliminated. However, this study has several limitations that need to be acknowledged. First, data on activity behavior rely primarily on self-reports, which may be subject to recall bias or social expectation bias. Second, the cross-sectional design of this study hinders the determination of causality. Third, potential measurement overlap exists between SST and sleep disorders, particularly regarding evening screen use affecting sleep quality, which may influence the precision of their individual associations with NSSI. Fourth, since the subjects of this survey were middle school students in urban areas of Hefei, the potential impact of urban–rural differences on the results was not considered. This may have a certain impact on the representativeness of our sample and further affect the extrapolation of the results. Future studies can be improved through longitudinal research, incorporating urban–rural difference analysis, and including objective measurement indicators such as accelerometers to further verify these findings.

## 5 Conclusion

The findings of this study indicate that NSSI is strongly associated with multiple sociodemographic characteristics and the allocation of time across 24-h activity behaviors. Prolonged ST and inappropriate time allocation of active behaviors are identified as significant risk factors for NSSI. According to the compositional logistic regression model analysis, increasing the duration of LPA and SLP is associated with a decreased risk of NSSI, while increasing the duration of ST and NSST is associated with an increased risk of NSSI. Furthermore, the CISM analysis revealed that substituting ST with LPA or SLP time could significantly reduce the risk of NSSI. For public health and school-based practice, this study highlights the need to prioritize balanced daily schedules and reduce prolonged sedentary time. Specific interventions should focus on promoting light physical activity and ensuring adequate sleep through structured routines, environmental adjustments, and behavioral substitution strategies. Targeted support for at-risk adolescents can also be integrated into school health programs.

## Data Availability

The raw data supporting the conclusions of this article will be made available by the authors, without undue reservation.
