# Supplementary material for: Association between screen time and non-suicidal self-injury among adolescents: a compositional isotemporal substitution analysis
Source: Front Public Health. 2026 Mar 11;14:1737730. doi: 10.3389/fpubh.2026.1737730 (PMC13013422; doi:10.3389/fpubh.2026.1737730)
Supplement: Supplementary file 1 [file Data_Sheet_1.docx]

Supplementary table 1 NSSI detection rates among adolescents with different ST durations

|  | N | NSSI | | 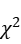 | *P* value |
| --- | --- | --- | --- | --- | --- |
|  |  | No (%) | Yes (%) |  |  |
| **Boy** |  |  |  | 11.40 | 0.001 |
| ＜120min/d | 843 | 687 (81.50) | 156 (18.50) |  |  |
| ≥120min/d | 2600 | 1973 (75.90) | 627 (24.10) |  |  |
| **Girl** |  |  |  | 14.64 | ＜0.001 |
| ＜120min/d | 682 | 501 (73.50) | 181 (26.50) |  |  |
| ≥120min/d | 2186 | 1434 (65.60) | 752 (34.40) |  |  |
| **Total** |  |  |  | 26.34 | ＜0.001 |
| ＜120min/d | 1525 | 1188 (77.90) | 337 (22.10) |  |  |
| ≥120min/d | 4786 | 3407 (71.19) | 1379 (28.81) |  |  |

NSSI: Non-Suicidal Self-Injury

Supplementary table 2 Geometric means of components used by adolescents at 24-hour times

|  | MVPA/min | LPA/min | SLP/min | ST/min | NSST/min |
| --- | --- | --- | --- | --- | --- |
| Boy  (N=3443) | 57.16  (3.97%) | 31.87  (2.21%) | 663.78  (46.10%) | 313.31  (21.76%) | 373.89  (25.96%) |
| Girl  (N=2868) | 32.61  (2.26%) | 32.42  (2.25%) | 652.75  (45.33%) | 328.38  (22.80%) | 393.86  (27.35%) |
| Total  (N=6311) | 44.35  (3.08%) | 32.16  (2.23%) | 659.63  (45.81%) | 320.50  (22.26%) | 383.36  (26.62%) |

MVPA: moderate-to-vigorous physical activity; LPA: low-intensity physical activity; ST: screen time; NSST: non-sedentary screen time; SLP: sleep

Supplementary table 3 NSSI detection rates among adolescents with different levels of physical activity and SLP duration

| Activity (min) | N | NSSI | | 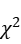 | *P value* |
| --- | --- | --- | --- | --- | --- |
|  |  | No (%) | Yes (%) |  |  |
| MVPA |  |  |  | 45.03 | ＜0.001 |
| ≤60 | 4154 | 2912 (70.10) | 1242 (29.90) |  |  |
| ＞60 | 2157 | 1683 (78.03) | 474 (21.97) |  |  |
| LPA |  |  |  | 0.73 | 0.394 |
| 180-240 | 83 | 57 (68.67) | 26 (31.33) |  |  |
| ＜180/＞240 | 6228 | 4538 (72.86) | 1690 (27.14) |  |  |
| SLP |  |  |  | 32.82 | ＜0.001 |
| 480-600 | 714 | 584 (81.79) | 130 (18.21) |  |  |
| ＜480/  ＞600 | 5597 | 4011 (71.66) | 1586 (28.34) |  |  |

MVPA: moderate-to-vigorous physical activity; LPA: low-intensity physical activity; SLP: sleep
