# Supplementary figures and images for: Association between screen time and non-suicidal self-injury among adolescents: a compositional isotemporal substitution analysis
Source: Front Public Health. 2026 Mar 11;14:1737730. doi: 10.3389/fpubh.2026.1737730 (PMC13013422; doi:10.3389/fpubh.2026.1737730)

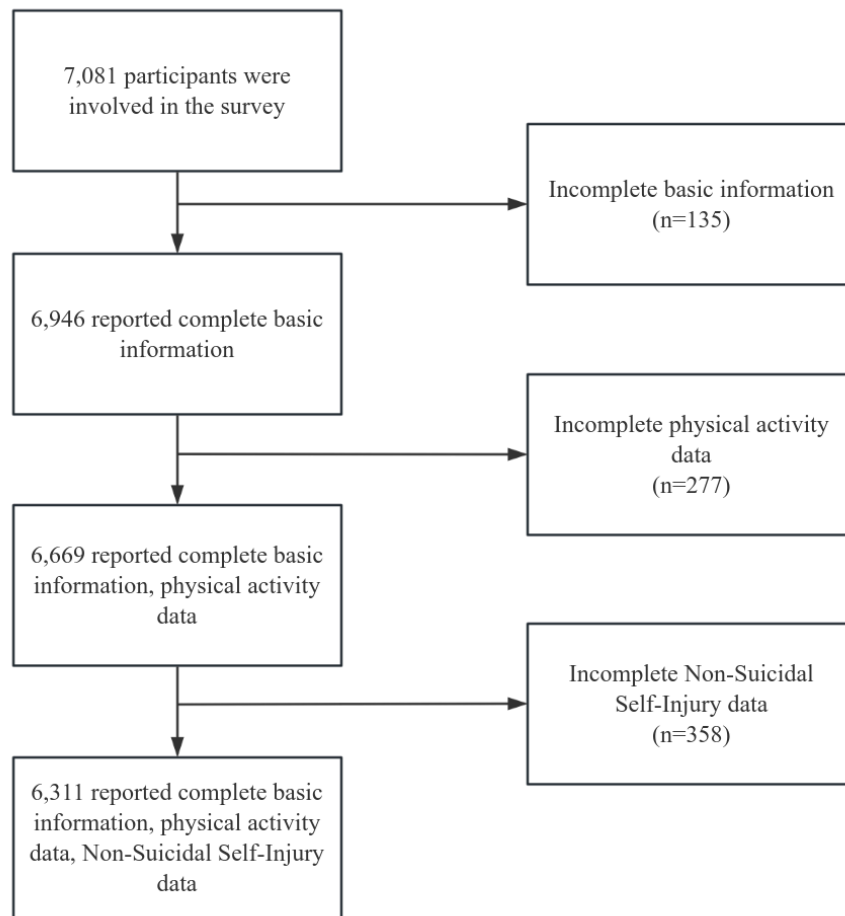

Supplementary Figure 1 Flowchart of participant inclusion

Supplement: Supplementary file 2 [file Image_1.pdf]
